# Demographics and Geographic Accessibility of Invasive Fungal Infection Clinical Trial Sites

**DOI:** 10.1001/jamanetworkopen.2026.17927

**Published:** 2026-06-11

**Authors:** Lucy X. Li, Jiashu Xue, Olivia S. Kates, Robin K. Avery, Sean X. Zhang, John W. Baddley, Christine M. Durand, Nitipong Permpalung

**Affiliations:** 1 Division of Infectious Diseases, Department of Internal Medicine, Johns Hopkins School of Medicine, Baltimore, Maryland; 2 CareDX, Brisbane, California; 3 Department of Pathology, Johns Hopkins School of Medicine, Baltimore, Maryland

## Abstract

This cross-sectional study investigates the geographic distribution and accessibility of clinical trials of systemic treatments for invasive fungal infections.

## Introduction

Clinical trials require substantial resources and infrastructure, contributing to regional variation in trial availability. Differential access can lead to disparities, particularly among socioeconomically disadvantaged and rural groups. These challenges are widely recognized in oncology^1^; however, disparities in invasive fungal infections (IFIs) have not been comprehensively examined, despite evidence that environmental factors strongly modify fungal disease risk.^2^ We hypothesized that US populations within reasonable travel distance of systemic antifungal trial sites would be more socioeconomically advantaged and less rural. Here, we investigate the distribution of these trials and the demographics of local populations.

## Methods

This cross-sectional study was deemed exempt from review by the Johns Hopkins institutional review board because it used publicly available, deidentified data. This study followed STROBE reporting guidelines.

Systemic antifungal trials registered on ClinicalTrials.gov from June 1, 2015, to June 1, 2025, were identified, excluding phase I. US trial site ZIP codes were mapped to ZIP code tabulation areas (ZCTAs) using Health Resources and Services Administration crosswalk, and a 30-mile buffer from each trial site ZCTA centroid was generated. This distance threshold aligns with common state health network adequacy standards and represents a pragmatic balance for geographic access.^3,4^ Socioeconomic and racial demographics were calculated using 2017 to 2021 American Community Survey (ACS) and 2020 Rural-Urban Commuting Area data,^5,6^ which rely on self-reported classifications. For buffer-level estimates, population was assumed to be uniformly distributed within each ZCTA and area-weighted accordingly. SEs for derived ACS estimates were propagated using US Census recommended formulas.^7^ Analyses were stratified by trial type (prophylaxis vs treatment). Global standardized mean differences (SMDs) were calculated to quantify the magnitude of differences for categorical variables (small, 0.2; medium, 0.5; large, 0.8). RStudio version 2025.09.0 + 387 was used for analysis.

## Results

Twenty-seven antifungal clinical trials (21 treatment, 6 prophylaxis) were identified across 249 unique sites, with a median (IQR) enrollment of 88 (31-199) participants per trial. Trial distribution varied by region; the South Atlantic division was notably overrepresented relative to total population (Figure, A). Within divisions, sites were unevenly distributed among and within states, particularly for prophylaxis trials (Figure, B and C).

**Figure.**
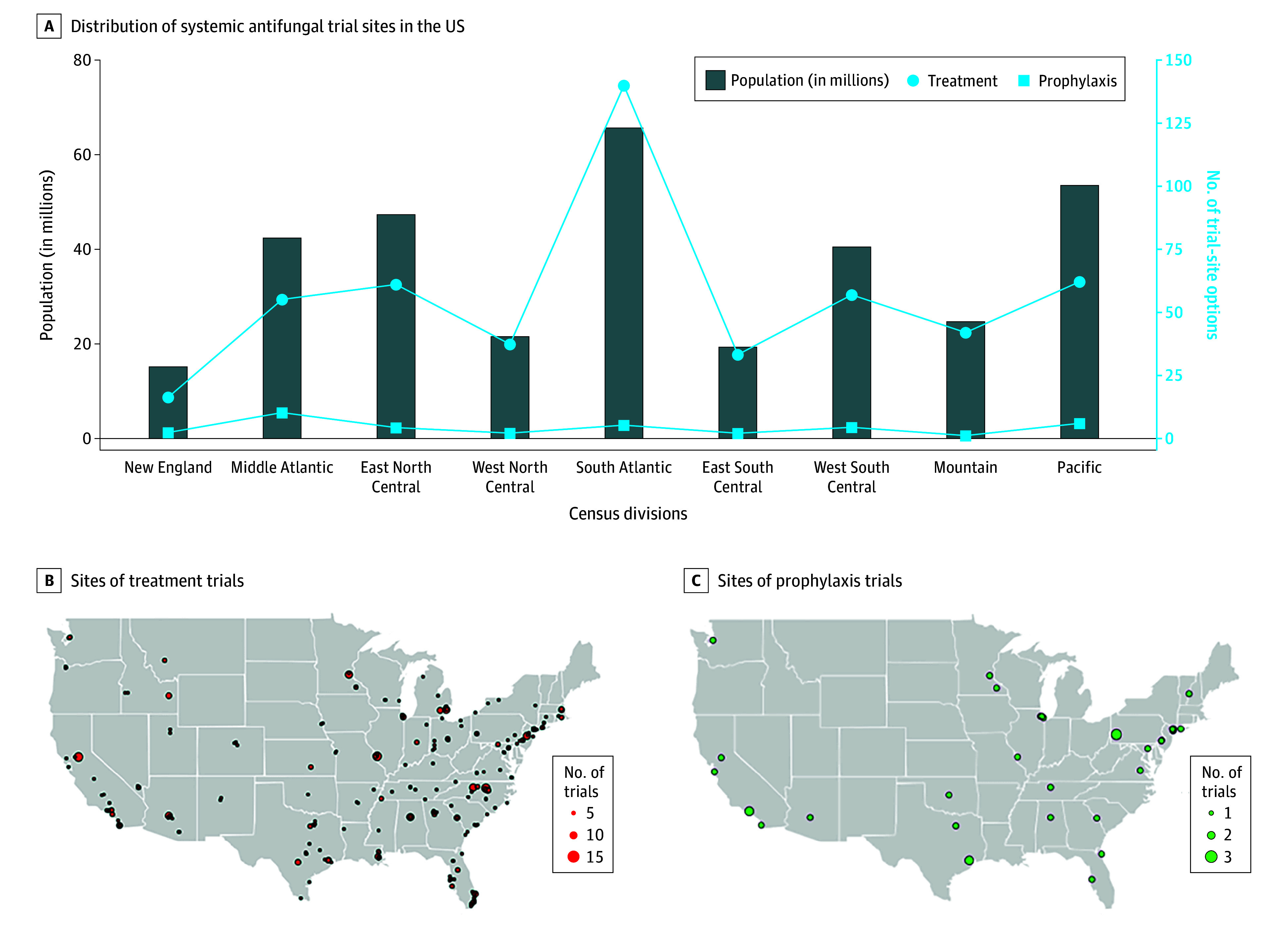
Bar Graph and Maps of Systemic Antifungal Trial Site Distribution in the US Treatment trials included 21 trials across 244 unique sites. Prophylaxis trials included 6 trials across 32 unique sites.

Trial sites were concentrated in urban areas; directly around treatment and prophylaxis sites, 99.3% and 98.6% of the population, respectively, resided in metropolitan areas with 0% in rural or small-town areas (Table). Expanding to a 30-mile radius, only 0.6% and 0.2% of the populations surrounding treatment and prophylaxis sites were rural, representing 7.2% and 1.1% of the total US rural population, respectively.

**Table.** Demographics of Individuals Residing in the Immediate and 30-Mile Area Around Systemic Antifungal Clinical Trial Sites

Characteristic	Total US		ZCTAs of trial sites						30 miles of trial sites			
			Tx			Ppx			Tx		Ppx	
	No. (SE), 10^3^	%	No. (SE), 10^3^	%	No. (SE), 10^3^		%	No. (SE), 10^3^		%	No. (SE), 10^3^	%
Age, y												
<18	74 830.7 (44.9)	22.5	1410.0 (6.5)	19.8	132.0 (2.1)		17.8	45 993.4 (36.7)		22.6	21 786.3 (25.3)	22.3
18-64	204 610.1 (70.5)	61.4	4639.9 (11.3)	65.2	508.5 (4.0)		68.6	126 943.9 (57.4)		62.3	61 480.5 (40.0)	63.0
≥65	53 592.1 (33.0)	16.1	1068.0 (4.9)	15.0	100.8 (1.7)		13.6	30 735.8 (25.8)		15.1	14 291.3 (17.8)	14.6
Global SMD	NA	NA	0.11	NA	0.20		NA	0.03		NA	0.05	NA
Race												
American Indian and Alaska Native	2727.5 (10.8)	0.8	37.3 (1.7)	0.5	4.1 (0.6)		0.6	1100.0 (8.1)		0.5	550.9 (5.9)	0.6
Asian	18 789.1 (30.8)	5.6	540.6 (5.1)	7.6	71.8 (1.9)		9.7	14 847.0 (27.8)		7.3	10 037.6 (22.5)	10.3
Black	41 722.4 (49.7)	12.5	1148.3 (8.4)	16.1	201.8 (3.5)		27.2	30 293.6 (43.8)		14.9	14 533.0 (30.6)	14.9
Native Hawaiian and Pacific Islander	615.7 (6.6)	0.2	8.4 (0.7)	0.1	0.5 (0.2)		0.1	291.6 (4.5)		0.1	161.9 (3.4)	0.2
White	226 480.5 (84.4)	68.0	4455.0 (12.7)	62.6	349.1 (3.8)		47.1	128 508.5 (66.7)		63.1	56 409.4 (45.4)	57.8
2 or More	23 600.1 (46.0)	7.1	556.4 (7.0)	7.8	47.5 (2.0)		6.4	15 132.3 (38.2)		7.4	7515.1 (27.3)	7.7
Other^a^	19 097.4 (40.6)	5.7	371.8 (5.8)	5.2	66.5 (2.7)		9.0	13 500.2 (34.6)		6.6	8350.2 (26.9)	8.6
Global SMD	NA	NA	0.19	NA	0.61		NA	0.15		NA	0.28	NA
Urbanicity												
Metropolitan	276 468.5 (93.1)	83.0	7069.3 (15.5)	99.3	731.3 (5.7)		98.6	197 350.0 (80.0)		96.9	96 866.8 (55.9)	99.3
Micropolitan	28 050.6 (20.8)	8.4	48.6 (0.3)	0.7	10.1 (0.3)		1.4	3756.4 (6.7)		1.8	373.4 (2.3)	0.4
Small town	8639.4 (10.9)	2.6	0	NA	0		NA	657.7 (2.8)		0.3	71.4 (0.6)	0.1
Rural	17 564.7 (18.3)	5.3	0	NA	0		NA	1269.2 (4.8)		0.6	188.5 (1.6)	0.2
Mixed^b^	2305.1 (6.7)	0.7	0	NA	0		NA	623.9 (3.6)		0.3	58.0 (0.8)	0.1
Global SMD	NA	NA	0.82	NA	0.78		NA	0.48		NA	0.65	NA
Language spoken at home												
English only	243 260.5 (84.5)	77.6	4867.9 (12.9)	72.4	493.1 (4.5)		70.1	141 475.4 (68.1)		73.9	62 129.6 (46.0)	67.7
Spanish												
English “very well”	25 677.2 (36.7)	8.2	631.9 (5.7)	9.4	67.3 (2.1)		9.6	17 818.5 (30.8)		9.3	9734.8 (22.6)	10.6
English less than “very well”	18 499.2 (30.1)	5.9	451.6 (5.0)	6.7	44.7 (1.7)		6.4	11 744.2 (25.3)		6.1	6689.0 (18.9)	7.3
Other												
English “very well”	16593.5 (29.8)	5.3	513.4 (5.1)	7.6	66.5 (1.8)		9.5	12921.7 (26.5)		6.7	8123.1 (20.8)	8.9
English less than “very well”	9457.2 (22.2)	3.0	260.8 (3.7)	3.9	31.4 (1.1)		4.5	7580.1 (19.8)		4.0	5070.7 (16.0)	5.5
Global SMD	NA	NA	0.21	NA	0.28		NA	0.12		NA	0.18	NA
Educational attainment												
Less than HS diploma	25 569.1 (28.8)	11.2	475.8 (4.1)	9.8	57.9 (1.5)		11.8	15 201.4 (22.8)		10.9	7853.8 (16.4)	11.7
HS diploma or equivalent	60 308.2 (41.5)	26.5	994.3 (5.7)	20.4	94.7 (1.9)		19.2	33 104.1 (32.1)		23.7	14 975.5 (21.7)	22.3
More than HS diploma	141 671.7 (61.7)	62.2	3400.7 (10.0)	69.8	340.0 (3.4)		69.0	91 125.0 (50.4)		65.4	44 378.6 (35.1)	66.0
Global SMD	NA	NA	0.18	NA	0.14		NA	0.09		NA	0.11	NA
Employment status^c^												
In labor force	169 106.0 (64.2)	63.4	3811.0 (10.3)	65.0	393.7 (3.5)		63.3	106 987.8 (52.4)		65.6	51 973.1 (36.5)	66.4
Civilian	167 907.1 (63.9)	62.9	3794.0 (10.3)	64.7	391.9 (3.5)		63.0	106 369.3 (52.2)		65.2	51 747.3 (36.3)	66.1
Employed	158 565.5 (61.7)	59.4	3584.4 (9.9)	61.1	362.1 (3.3)		58.2	100 463.1 (50.2)		61.6	48 752.3 (34.9)	62.3
Unemployed	9341.6 (17.7)	3.5	209.7 (2.7)	3.6	29.8 (1.0)		4.8	5906.2 (14.4)		3.6	2994.9 (10.2)	3.8
Military	1198.9 (7.7)	0.4	17.0 (0.8)	0.3	1.8 (0.4)		0.3	618.5 (5.9)		0.4	225.8 (4.0)	0.3
Not in labor force	97 776.3 (49.7)	36.6	2051.3 (7.8)	35.0	228.7 (2.7)		36.7	55 990.2 (39.2)		34.4	26 294.8 (27.0)	33.6
Global SMD	NA	NA	0.08	NA	0.06		NA	0.07		NA	0.08	NA
Ratio income to federal poverty level												
<0.5	19 342.3 (36.9)	5.9	509.8 (5.9)	7.4	76.5 (2.4)		11.1	11 006.6 (29.1)		5.5	5188.5 (20.1)	5.4
0.5 to <1.0	22 719.5 (42.4)	7.0	520.7 (6.5)	7.6	68.7 (2.4)		10.0	12 741.9 (33.3)		6.4	5951.0 (23.0)	6.2
1.0 to <1.5	26 641.5 (47.5)	8.2	543.3 (6.6)	7.9	54.5 (2.3)		7.9	14 969.4 (37.3)		7.5	6845.6 (25.4)	7.1
1.5 to <2.0	27 685.0 (48.0)	8.5	552.0 (6.6)	8.1	52.5 (2.2)		7.6	15 704.1 (37.6)		7.9	7136.0 (25.7)	7.5
≥2.0	228 789.6 (94.2)	70.4	4728.2 (14.1)	69.0	436.4 (4.8)		63.4	145 398.6 (76.4)		72.8	70 754.4 (53.3)	73.8
Global SMD	NA	NA	0.17	NA	0.35		NA	0.10		NA	0.14	NA
Vehicles per person in household												
None	10 524.7 (15.9)	8.4	394.0 (3.0)	13.6	121.7 (1.8)		39.1	7472.6 (13.6)		9.8	4698.4 (10.6)	13.0
>0 to <1	43 661.0 (32.4)	34.9	959.7 (5.0)	33.2	86.5 (1.6)		27.8	27 679.4 (26.1)		36.2	13 512.7 (18.0)	37.5
≥1	71021.4 (39.8)	56.7	1535.5 (6.2)	53.1	103.3 (1.5)		33.2	41 211.7 (31.0)		54.0	17 830.0 (20.2)	49.5
Global SMD	NA	NA	0.17	NA	0.73		NA	0.06		NA	0.18	NA

Abbreviations: HS, high school; NA, not applicable; Ppx, prophylaxis; SMD, standardized mean differences comparing population distribution of each area to that of total US; Tx, treatment; ZCTA, ZIP code tabulation area.

^a^ Other, self-reported as not identifying with US Census Bureau race classifications.

^b^ Mixed, ZCTAs with multiple Rural-Urban Community Area classifications.

^c^ Among individuals of working age defined as aged ≥16 years.

Demographically, most residents in both immediate (treatment, 65.2%; prophylaxis, 68.6%) and extended (treatment, 62.3%; prophylaxis, 63.0%) catchments were of working age. Racial minority groups were more highly represented in immediate and extended catchments of prophylaxis sites (SMD ZCTA, 0.61; 30 miles, 0.28) compared with the US population, while treatment sites were more similar to the US population (SMD ZCTA, 0.19; 30 miles, 0.15) (Table).

Socioeconomically, the distribution of residents among labor force categories was comparable between the immediate (SMD treatment, 0.08; prophylaxis, 0.06) and extended (SMD treatment, 0.07; prophylaxis, 0.08) catchment areas and the overall US population. Additionally, a higher proportion of residents with incomes below 150% of the federal poverty level resided in the immediate catchment areas compared with the overall US population (SMD treatment, 0.17; prophylaxis, 0.35); however, the extended catchment areas were comparable to the US (SMD treatment, 0.10; prophylaxis, 0.14). A substantial proportion of trial ZCTA residents had fewer vehicles than household members or had no vehicle (treatment, 46.8%; prophylaxis, 66.9%), and vehicle availability remained limited in the extended catchment area (treatment, 46.0%; prophylaxis, 50.5%) (Table).

## Discussion

IFI trial sites are concentrated in metropolitan locations, potentially leading to underrepresentation of IFI epidemiologic diversity in regions with less research activity but significant IFI burden (eg, fungicide-exposed rural areas). With the expanding geographic range of IFIs and differential incidence patterns between urban and rural settings (eg, higher rates of histoplasmosis in rural regions),^8,9^ this underrepresentation may perpetuate data disparities and impact true effect estimation in trials, especially for prophylaxis.

Proximity also does not ensure representative enrollment. Individuals with lower income, education, or English proficiency are more likely to reside or work in settings with higher mold exposure yet have more participation barriers.^2^ Participation requires financial capacity, social resources, and scientific engagement, even when direct costs are defrayed.^10^

This study was limited by lack of participant-level data, precluding analysis of trial demographics and indirect costs. The descriptive approach provided a foundational characterization of geographic access; future studies using spatial regression methods could extend this work to inform equitable trial network planning.

The nuanced interplay of participation barriers with environmental and socioeconomic risk factors emphasizes the complexity of representative enrollment. These findings highlight the need for strategies, eg, expanded trial network or registry, to ensure equitable participation and access and better capture disease diversity to advance antifungal drug development.

## Notes

Supplement.
Data Sharing Statement
