# Supplementary material for: Demographics and Geographic Accessibility of Invasive Fungal Infection Clinical Trial Sites
Source: JAMA Netw Open. 2026 Jun 11;9(6):e2617927. doi: 10.1001/jamanetworkopen.2026.17927 (PMC13261487; doi:10.1001/jamanetworkopen.2026.17927)
Supplement: Supplement. — Data Sharing Statement [file jamanetwopen-e2617927-s001.pdf]

Data Sharing Statement

Li. Demographics and Geographic Accessibility of Invasive Fungal Infection Clinical Trial Sites. *JAMA Netw Open*. Published June 11, 2026. doi:10.1001/jamanetworkopen.2026.17927

Data
Data available: Yes
Data types: Other (please specify)
Additional Information: All data regarding the clinical trials included in this study are already publicly available.
How to access data: The data used for this study are publicly available from https://www.census.gov/ and https://clinicaltrials.gov/.
When available: With publication

Supporting Documents
Document types: Statistical/analytic code
How to access documents: Analytic code is available from Dr. Li (email, lli101@jh.edu) upon request.
When available: With publication

Additional Information
Who can access the data: The data is already publicly available, and the analytic code will be made available to anyone requesting the data.
Types of analyses: The data is already publicly available, and the analytic code will be made available for any purpose.
Mechanisms of data availability: Code is available upon request with investigator support.
